# A Comprehensive Membrane Process for Preparing Lithium Carbonate from High Mg/Li Brine

**DOI:** 10.3390/membranes10120371

**Published:** 2020-11-26

**Authors:** Wenhua Xu, Dongfu Liu, Lihua He, Zhongwei Zhao

**Affiliations:** School of Metallurgy and Environment, Central South University, Changsha 410083, China; xuwenhua@csu.edu.cn (W.X.); liudongfu@csu.edu.cn (D.L.)

**Keywords:** membrane process, Li_2_CO_3_, electrochemical intercalation deintercalation, high Mg/Li brine

## Abstract

The preparation of Li_2_CO_3_ from brine with a high mass ratio of Mg/Li is a worldwide technology problem. Membrane separation is considered as a green and efficient method. In this paper, a comprehensive Li_2_CO_3_ preparation process, which involves electrochemical intercalation-deintercalation, nanofiltration, reverse osmosis, evaporation, and precipitation, was constructed. Concretely, the electrochemical intercalation-deintercalation method shows excellent separation performance of lithium and magnesium, and the mass ratio of Mg/Li decreased from the initial 58.5 in the brine to 0.93 in the obtained lithium-containing anolyte. Subsequently, the purification and concentration are performed based on nanofiltration and reverse osmosis technologies, which remove mass magnesium and enrich lithium, respectively. After further evaporation and purification, industrial-grade Li_2_CO_3_ can be prepared directly. The direct recovery of lithium from the high Mg/Li brine to the production of Li_2_CO_3_ can reach 68.7%, considering that most of the solutions are cycled in the system, the total recovery of lithium will be greater than 85%. In general, this new integrated lithium extraction system provides a new perspective for preparing lithium carbonate from high Mg/Li brine.

## 1. Introduction

The fast development of electric vehicles, storage devices, and hand-held electronic devices has dramatically increased the demands for lithium [1,2,3,4]. Lithium carbonate is an important raw material for preparing lithium-ion battery cathode materials [5]. In recent years, global lithium (Li) demand has reached 180,000 tons of lithium carbonate equivalent in 2015, with forecasts as high as 1.6 M tons by 2030 [6,7].

Nowadays, lithium resources mainly exist in solid ore (such as spodumene and lepidolite) and brine, and over 70% of exploitable lithium in the world existed in the brine [8,9]. Compared with the lithium extraction from these two kinds of resources, lithium extraction from brine is more effective, simpler, and cheaper [8]. Most lithium resources in continental brines are found in a small region in South America, often referred to as the “Lithium Triangle” [9,10]. A notable feature of brines in the “Lithium Triangle” region is the low mass ratio of Mg/Li. In contrast, the grade of brine in other regions is much worse. In China, the major lithium-containing brines are located in the Qinghai–Tibet plateau [8,11], and most of the lithium-containing brines in this area are mostly magnesium sulfate subtype [12]. A typical feature of magnesium sulfate subtype brines is the mass ratio of Mg/Li, which has a long span (from tens to hundreds, even more than 1000) [13]. Therefore, how to effectively realize the separation of magnesium and lithium is the key to produce Li_2_CO_3_ from high Mg/Li brines.

Multifarious methods such as solvent extraction [14], membrane separation [15,16,17], adsorption [18,19], and electrochemical intercalation-deintercalation (EID) method [17,20,21,22,23] have been developed for lithium extraction from high Mg/Li brine. Solvent extraction is an efficient separation technology; both of the separation factors (SF_Li–Na_, SF_Li–Mg_) can reach hundreds or even more than one thousand [24,25]. However, the extraction reagent has a slight solubility in aqueous solution [26], which is not suitable for treating brine directly. The ion-sieve absorption method is considered to be an effective approach to extract lithium from the high Mg/Li ratio brines thanks to its low cost, high selectivity, and nontoxicity [27]. However, the ion-sieve absorption method faces the following problems: (1) it is difficult to prepare the high absorption capacity absorbent; and (2) there is a significant loss of capacity in the desorption process when acids or oxidants are used as desorption agents. The above problems seriously restrict its large-scale industrial application [20].

Nanofiltration (NF), as an important membrane separation technology, has been successfully applied for separating lithium and magnesium from a high Mg/Li brine because of its selective rejection of divalent ions and monovalent ions based on Donnan exclusion [28,29]. However, it also suffers from the following problems: (1) This technology can only treat brine with very low sodium and potassium content, and it usually takes 1–2 years to obtain this kind of brine [30,31,32]. (2) The salinity in the type of brine after potassium removal is too high to meet the operation condition for this purpose, which needs to be diluted with water (the amount of water used for dilution is usually several times than the brine). This process not only needs to consume a large amount of fresh water, but also increases the amount of water to be treated.

In our previous work, we have proved that the EID method shows an excellent lithium extraction properties from the high mass ratio of brine [20,21,33]; the mass ratio of brine can be decreased from the initial 58.5 in the brine to 0.93 in the obtained anolyte. Although the mass ratio of Mg/Li in the anolyte is much lower than the original brine, the lithium concentration in the anolyte is only 1–2 g·L^−1^, which is far from the lithium concentration required to precipitate lithium carbonate. For this reason, we need to concentrate the anolyte and remove the residual impurities (e.g., Mg^2+^, Ca^2+^, and SO_4_^2−^) in it. Theoretically, all kinds of concentration methods (like reverse osmosis, electrodialysis, evaporation, and so on) [34,35] and impurity removal methods (like nanofiltration, solvent extraction, and so on) [24,28,29] can be used to treat the obtained anolyte. Notably, the total salt concentration of the obtained anolyte is between 20 and 30 g·L^−1^, which is an ideal range for NF and reverse osmosis (RO) treatment. Therefore, we proposed an integrated lithium carbonate preparation process combining EID, NF, RO, evaporation, and precipitation processes to prepare Li_2_CO_3_ from a high Mg/Li brine. The aim of the main processes are as follows: (1) the EID method is used to maximize the separation of magnesium and lithium from the brine to obtain a low Mg/Li anolyte; (2) removing the multivalent ions (e.g., Mg^2+^, Ca^2+^, and SO_4_^2−^) from the obtained anolyte via the NF method; (3) concentrating the permeate flow produced by NF with the RO method; (4) further increasing the lithium concentration by evaporation; and (5) precipitating Li_2_CO_3_ by adding Na_2_CO_3_.

## 2. Materials and Methods

### 2.1. Membranes

The membrane used in the EID method is a heterogeneous anionic membrane (MA-3475), which was purchased from Beijing Anke Membrane Separation Technology & Engineering Co., LTD. (Beijing, China agent). The heterogeneous anionic membrane selectively allows the anions to pass through and reject the cations. The NF (NF2) and RO (RO5) membranes used for the experiment are disc tube membranes, which were made by RisingSun Membrane Technology Co., Ltd., (Beijing, China). Specifically, the membrane areas of the NF membrane and RO membrane are both 2.2 m^2^, and the operation pH are in the range of 3–11. The permeate flux and desalination rate of NF were 42 L·m^−2^·h^−1^ and 98%, respectively, which were obtained at 25 °C, operating pressure of 0.7 MPa, and test salt concentration of MgSO_4_ of 2 g·L^−1^. Further, the permeate flux and desalination rate of RO were 42 L·m^−2^·h^−1^ and 99.5%, respectively, which were obtained at 25 °C, operating pressure of 1.55 MPa, and test salt concentration of NaCl of 2 g·L^−1^.

### 2.2. Experimental Illustration

#### 2.2.1. Methods

LiFePO_4_/FePO_4_ electrodes’ preparation: LiFePO_4_ electrode was prepared as follows: (1) weighing LiFePO_4_, polyvinylidene fluoride (PVDF), and acetylene black (C) in a mass ratio of 8:1:1; (2) dissolving PVDF into N-methylpyrrolidone (NMP) and then adding C and LiFePO_4_ in order; (3) coating the above-mixed slurry on a carbon fiber sheet; and (4) drying the prepared carbon fiber sheet in a vacuum oven at 95 °C for 12 h. The FePO_4_ electrode was obtained by deintercalating lithium from the LiFePO_4_ electrode. Concretely, an electrolytic cell is divided into an anode chamber and cathode chamber by anion membrane, LiFePO_4_ electrode (anode) and nickel foam (cathode) were placed into the anode and cathode chamber, respectively. Both of the chambers were filled with 5 g·L^−1^ NaCl solution and the pH value of the catholyte was controlled to 2–3 using HCl. The voltage used in electrolysis is 1.0 V, and the electrolysis ends until the current density is less than 0.05 mA·cm^−2^.

EID method for lithium extraction: The device for the EID method is shown in our previous work [20]. The device of the EID system was divided into two chambers by the anion membrane, where LiFePO_4_ and FePO_4_ is used as anode and cathode, respectively. The anode and cathode chambers are filled with supporting electrolyte and brine, respectively. The entire working process is shown below: (1) lithium deintercalated from LiFePO_4_ to the supporting electrolyte (LiFePO_4_ – e = Li^+^ + FePO_4_); (2) lithium existed in the brine intercalated into FePO_4_ (Li^+^ + FePO_4_ + e = LiFePO_4_); and (3) the Cl^−^ in the brine diffused into the anode chamber through the anionic membrane to maintain the electroneutrality of the anolyte and brine, and LiCl was obtain in the anolyte.

The brine used for the lithium extraction was from West Taijnar Salt Lake (Golmud, China) with the Mg/Li ratio of 58.8 (Table 1), and the lithium extraction process was carried out via instrument LANHE-CT2001A (Wuhan, China). The effective size of the electrodes was 17 × 20 cm^2^, and the electrodes of LiFePO_4_ and FePO_4_ worked as anode and cathode, respectively. The electrode coating density was about 85 mg (LiFePO_4_)·cm^−2^. The electrolytic cell was comprised of two chambers, which were separated by an anion exchange membrane (MA-3475, Beijing Anke Membrane Separation Technology Engineering Co. LTD, Beijing, China agent). The anode chamber was filled with 1.5 L of 5 g·L^−1^ NaCl as supporting electrolyte, and the cathode chamber was filled with 1.5 L brine. The entire electrolysis was performed with a constant current of 0.6 A until the voltage reached 0.35 V, and then worked at a constant voltage until the current dropped to 0.1 A to end the electrolysis process.

NF for purification: The obtained anolyte was purified by NF, the volume of the feed used in the nanofiltration was 200 L, and the composition of the feed used was configured according to the composition of the anolyte obtained by the EID system. The NF process was carried out at a constant pump power of 2 KW until the operation pressure reached 8 MPa. The total volume of the collected permeate solution was 180 L.

RO for concentration: The collected permeate solution after the NF treatment was concentrated by RO process, and only 175 L feed liquor was used in the process. The whole RO process was performed at the room temperature and ended until the volume of permeate reached 105 L. The RO process was also carried out at a constant pump power of 2 KW.

Evaporation and concentration: The evaporation process was carried out by an electric furnace, and the initial volume of the solution used for the evaporation was 5 L.

Precipitation of Li_2_CO_3_: The lithium-containing solution after evaporation process was precipitated by Na_2_CO_3_ (280 g·L^−1^) at 95 °C. The addition of sodium carbonate is 1.05 times the dosage of theoretical amount used in the lithium precipitation reaction. When all of the Na_2_CO_3_ wass added into the LiCl solution, the solution was stirred for 1 h to mature the lithium carbonate, and then the Li_2_CO_3_ was filtered out. The obtained lithium carbonate was washed twice with deionized water and dried to obtain the Li_2_CO_3_ product.

**Figure 1:**
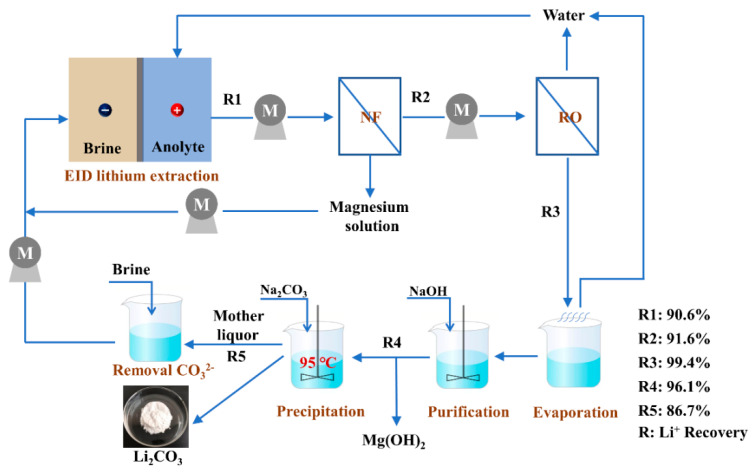
Schematic diagram of the comprehensive membrane process. EID, electrochemical intercalation-deintercalation; NF, nanofiltration; RO, reverse osmosis.

In general, the comprehensive membrane process is shown in Figure 1.

#### 2.2.2. Analytical Methods

The concentration of Li^+^, Na^+^, K^+^ and Mg^2+^, and Ca^2+^ in the solutions was measured by inductively coupled plasma-optical emission spectrometry (ICP-OES, Thermo Scientific iCAP-7200, Shanghai, China agent), and the concentration of SO_4_^2−^ was measured by ion chromatography (ICS-5000/DIONEX, Thermofisher Scientific, Shanghai, China agent). The X-ray Diffraction (XRD) patterns were measured via a BRUKER D8 ADVANCE using Cu-Kα radiation (λ = 1.54056 Å). The morphology of Li_2_CO_3_ was detected by a scanning electron microscope (SEM, JEOL JSM-6490LV, JEOL (BEIJING) CO., LTD., Beijing, China agent).

#### 2.2.3. Calculation

The separation factor (SF) of lithium and magnesium was calculated as Equation (1):

(1) $$\mathrm{SF}=\frac{C_{\mathrm{Li}}/C_{\mathrm{Mg}}}{C_{\mathrm{Li}}^{\prime }/C_{\mathrm{Mg}}^{\prime }}$$

where SF is the separation factor of Li^+^ and Mg^2+^, *C*_Li_ is the concentration of lithium in the obtained solution (g·L^−1^), *C*_Mg_ is the concentration of magnesium in the obtained solution (g·L^−1^), $C_{\mathrm{Li}}^{\prime }$ is the concentration of lithium in the feed (g·L^−1^), and $C_{\mathrm{Mg}}^{\prime }$ is the concentration of magnesium retained in the feed (g·L^−1^).

The recovery of lithium (*R*_E_) for the electrolytic intercalation-deintercalation system was calculated as Equation (2):

(2) $$R_{E}=\frac{C_{0}V_{0}-\int_{0}^{t}C_{t}V_{t}}{C_{0}V_{0}}\times 100\%$$

where *R*_E_ is the recovery of lithium in the brine, *C*_0_ is the initial concentration of lithium in the brine (g·L^−1^), *V*_0_ is the initial volume of the brine (L), *t* is the sampling time (h), *C_t_* is the concentration of lithium in brine at *t* (g·L^−1^), and *V_t_* is the volume of brine at *t* (L).

The retention ratio (*R*) refers to the permeability of ions, which is the main index to evaluate the separation performance. The corresponding calculation process is shown in Equation (3).

(3) $$R=\frac{C_{F}V_{F}-C_{P}V_{P}}{C_{F}V_{F}}\times 100\%$$

where *R* represent the retention ratio and *C_F_* and *C_P_* are the concentrations of ions of the feed and permeate solution (g·L^−1^), respectively. *V_F_* and *V_P_* are the volume of the feed and permeate solution (L).

#### 2.2.4. Membrane Cleaning

The membranes need to be washed when the transmembrane pressure difference is greater than 0.35 MPa. For the membrane scaling caused by inorganic salts, 1% (wt) ethylenediamine tetraacetic acid disodium salt (EDTA) + citric acid solution (citric acid is used to adjust the pH of the solution to 3–4) is generally used for cleaning at room temperature for about 1 h.

## 3. Results and Discussion

### 3.1. Lithium Extraction From the Brine

**Figure 2:**
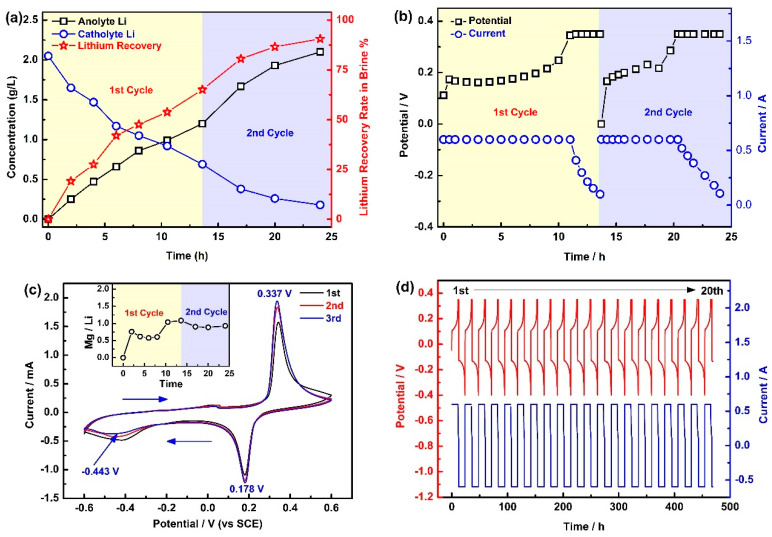
The EID system for lithium extraction. (**a**) Li^+^ concentration and Li^+^ recovery rate in the first two cycles; (**b**) current and voltage changes in two cycles; (**c**) the cyclic voltammetry (CV) curves of brine and the illustration shows the Mg/Li in the obtained anolyte; (**d**) charge and discharge cycle performance of the brine. SCE, saturated calomel electrode.

The primary contents of the West Taijinar used for the lithium extraction are shown in Table 1, and the experimental results are exhibited in Figure 2. From Figure 2a, it can be seen that the concentration of lithium reached 2.1 g·L^−1^ at the end of the second cycle, and the concentration of lithium in the brine decreased from the initial 2.05 g·L^−1^ to 0.18 g·L^−1^, while the total recovery of lithium reached 90.6% at the end of the second cycle. In the same way, the decline rate of lithium in the second cycle is slightly lower than that in the first cycle, which is mainly owing to the continuous decline of lithium concentration in the brine.

Figure 2b shows the voltage and current curves in the first two cycles. It can be seen that the first cycle took 13.5 h, while the second cycle only lasted 10.5 h. In addition, the constant current process in the first cycle lasts longer than in the second cycle. Correspondingly, the voltage growth rate in the first cycle is also slower. The above results are attributed to the fact that the lithium concentration in the second cycle is lower than that in the first cycle, which leads to more serious polarization of lithium extraction in the second cycle.

Figure 2c shows the cyclic voltammetry (CV) curves of LiFePO_4_ in the brine; it can be seen that there are a couple of obvious peaks for the deintercalation/intercalation of lithium located at 0.337 V (vs. saturated calomel electrode (SCE)) and 0.178 V (vs. SCE), which correspond to the deintercalation of lithium from LiFePO_4_ (LiFePO_4_ – e = Li^+^ + FePO_4_) and the intercalation of lithium to FePO_4_ (FePO_4_ + Li^+^ + e = LiFePO_4_), respectively. There also exists a weak reduction peak at −0.443 V (vs. SCE), which corresponds to the intercalation of magnesium (FePO_4_ + 0.5 Mg^2+^ + e = Mg_0.5_FePO_4_). Obviously, magnesium is more difficult to insert into FePO_4_ than lithium, which means that FePO_4_ can selectively extract lithium from a high Mg/Li brine via potential control. In addition, the inset illustration in Figure 2c shows that the mass ratio of Mg/Li in the obtained anolyte is only 0.93, which is far lower than 58.5 in the brine. The above results show that the new EID system has excellent separation performance for lithium and magnesium.

Figure 2d shows the charge/discharge curves of LiFePO_4_ in the West Taijinar brine. It can be seen that the charging and discharging curves of the 20 cycles are relatively stable, which means that LiFePO_4_ can operate stably in the brine.

Furthermore, the analysis results of the main ions in the produced anolyte are shown in Table 2. From Table 2, it can be seen that the main ions in the anolyte are Li^+^, Na^+^, and Mg^2+^. Compared with the Mg^2+^ concentration in the brine, the penetration of magnesium into the anolyte is negligible. The rejection rates of the impurities such as K^+^, Mg^2+^, and SO_4_^2−^ are 92.2%, 98.5%, and 99.2%, respectively. The retention of cations by the anion membrane is mainly due to the charge repulsion of the fixed cationic groups of the membrane itself to the cations in the solution [36,37]. The interception of divalent sulfate is mainly due to the fact that the ionic radius of sulfate is larger than that of chloride ions, and the concentration of chloride ions is much greater than that of sulfate, which makes the content of sulfate permeable through the membrane very low in the process of lithium extraction. In general, the concentration of the impurities in the obtained anolyte is very low, which is facilitation for the subsequent purification process.

Therefore, the EID system shows excellent separation properties of lithium and magnesium. It is an efficient, environmentally friendly, and stable process without using acid, alkali, or any toxic reagents, nor does it produce any solid waste. The brine after the lithium extraction can be directly discharged back to the salt fields, without affecting the environment.

### 3.2. NF and RO Processes

In order to precipitate lithium carbonate, the lithium-riched anolyte needs to be deeply purified and concentrated. In this paper, NF and RO were used for deep purifying of the divalent ions and concentrating of the penetrating fluid, respectively. Both NF and RO were carried out only once and the corresponding results of the NF and RO processes are shown in below.

#### 3.2.1. NF Process

**Figure 3:**
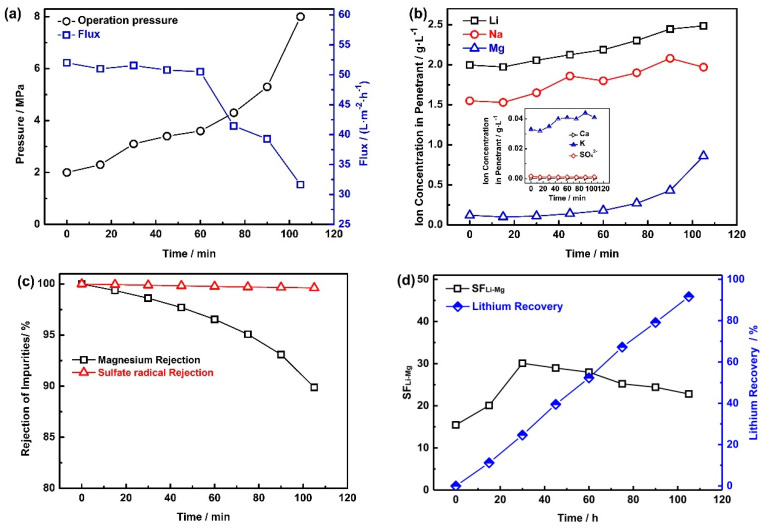
The NF process for purification. (**a**) The relationship of the operation pressure and the flux of the membrane; (**b**) concentration of Li^+^, Na^+^, K^+^, Mg^2+^, Ca^2+^, and SO_4_^2−^ in the permeate flow; (**c**) the rejection rate of Mg^2+^ and SO_4_^2−^; (**d**) the recovery of lithium and the separation factor of lithium and magnesium (SFLi-Mg).

The corresponding experimental results are shown in Figure 3 and Table 3. As shown in Figure 3a, the initial operation pressure of the nanofiltration was 2.0 MPa. The water flux decreases slowly from the initial 52 L·m^−2^·h^−1^ to 50.5 L·m^−2^·h^−1^ at first, and then rapidly declines from 50.5 L·m^−2^·h^−1^ to 31.6 L·m^−2^·h^−1^. Inversely, the operation pressure increases at first and then rapidly reaches 8 MPa. There are three main reasons for this phenomenon: (1) the increase of the osmotic pressure in the retentate solution; (2) the precipitation of the salts on the surface of the NF membrane; and (3) the compaction of the NF membrane.

The concentration of the ions in the permeate flow and collected retentate during the NF process are shown in Table 3 and Figure 3b, respectively. From Table 3, it can be seen that the conductivity increased slowly at the beginning of the initial stage (increased from 37.5 mS·cm^−1^ to 52.0 mS·cm^−1^). Subsequently, a significant increase followed after 90 min from the start of the NF, and the conductivity reached 108.2 mS·cm^−1^. The temperature rose slowly throughout the experiments (from 24.7 °C to 27.5 °C), and the rise in water temperature comes from two aspects: (1) the mechanical friction of the high pressure pump produce a great deal of heat; and (2) the friction of the fluid and the pipe, which also generates heat. The flow rate of the entire NF process was kept at 16 L·min^−1^. There was no significant change in the concentration of monovalent ions such as Li^+^, Na^+^, and K^+^, while divalent ions such as Mg^2+^, Ca^2+^, and SO_4_^2−^ are abundantly enriched in the retentate solution. Moreover, the concentration of Mg^2+^, Ca^2+^, and SO_4_^2−^ at the end of the NF process reached 18.23 g·L^−1^, 0.02 g·L^−1^, and 2.41 g·L^−1^, respectively. It can be found that Mg^2+^, Ca^2+^, and SO_4_^2−^ were concentrated 9.3 times, 5 times, and 9.1 times, respectively. The concentrated times of Ca^2+^ were lower than those of Mg^2+^ and SO_4_^2−^. Notably, the main anion in the collected retentate is Cl^−^, which was rejected to maintain the electrical neutrality of the collected retentate.

As shown in Figure 3b, the concentration of Li^+^, Na^+^, Mg^2+^, and K^+^ in the permeate flow increased obviously, while the concentration of Ca^2+^, and SO_4_^2−^ is very low and can almost be ignored (Ca^2+^ and SO_4_^2−^ are 3.1 × 10^−4^ g·L^−1^ and 1.07 × 10^−3^ g·L^−1^ at the end of the NF process). Specifically, the concentration of Li^+^ and Na^+^ increased from 2 g·L^−1^ to 2.54 g·L^−1^ and 1.55 g·L^−1^ to 1.97 g·L^−1^, respectively. Moreover, the concentration of K^+^ also increased slowly from 0.033 g·L^−1^ to 0.041 g·L^−1^. In contrast, the concentration of Mg^2+^ increased sharply from 0.12 g·L^−1^ to 0.86 g·L^−1^. Combining the concentration of ions (Li^+^, Na^+^, and Mg^2+^) in the collected retentate, it can be found that there is basically no interception of monovalent ions, while the interception rate of multivalent ions is very high. The reason for the higher rejection of Mg^2+^ can be explained using Donnan exclusion. The concentration of counter ions (ions with charge opposite to the fixed charge in the membrane) in the membrane is higher than that in the bulk solution, while the concentration of homonymous ions in the membrane is lower than that in the bulk solution. The Donnan difference prevents the diffusion of homonymic ions from the bulk solution into the membrane. In order to maintain electrical neutrality, the counter ions are also trapped by the membrane. The coulomb repulsion of the multivalent ions is greater than that of the monovalent ions, which explains why the rejection of Mg^2+^ is higher than that of Li^+^ and Na^+^.

The rejection rate of the divalent ions is shown in Figure 3c. It can be seen that the rejection rates of SO_4_^2−^ are higher than 99%, while the retention rates of magnesium gradually drop to 89.9%. Combining the data presented in Figure 3b, it can be found that the concentration of Ca^2+^ and SO_4_^2−^ in the permeate flow can almost be ignored, which means that sulfate and calcium ions can hardly pass through the nanofiltration membrane. In order to determine whether there is precipitation in the NF process, the solubility of all chlorides and sulfates in the solution at 20 °C is listed, as shown in Table 4.

According to the results provided by Table 3 and Table 4, all of the soluble salts that exist in the collected retentate are not saturated. Notably, there is 0.004 g·L^−1^ Ca^2+^ and 0.26 g·L^−1^ SO_4_^2^^−^ in the beginning of the NF, which results in 0.04 g·L^−1^ Ca^2+^ and 2.6 g·L^−1^ SO_4_^2−^ at an assumed retention of 100%, and the concentration of Ca^2+^ and SO_4_^2−^ has not reached the *K*_sp_ of CaSO_4_·2H_2_O (the solubility of CaSO_4_·2H_2_O is 0.255 g at 20 °C, which means the *K*_sp_ of CaSO_4_·2H_2_O is 2.2 × 10^−4^) [38]. Because of the retention of divalent ions by the NF membrane and the influence of the electric double layer, a large amount of divalent ions will be enriched on the surface of the NF membrane. When the sulfate and calcium in the bulk retentate solution have not reached the conditions for CaSO_4_·H_2_O precipitation, there is already CaSO_4_·H_2_O precipitation on the surface of the nanofiltration membrane. That is the reason the concentration of Ca^2+^ in the bulk collected retentate is only 0.02 g·L^−1^, as the feed solution has concentrated 10 times. Further, because the total amount of Ca^2+^ is much lower than that of SO_4_^2−^, this results in the lower concentrated times of Ca^2+^ than SO_4_^2−^. In order to reduce the membrane scaling caused by calcium sulfate precipitation, it is better to wash the membranes after the NF operation. By contrast, Mg^2+^ can only be continuously accumulated in the collected retentate without precipitation, resulting in a higher concentration of Mg^2+^ in the permeate flow. In other words, the more Mg^2+^ that enters the permeate flow, the lower the retention rate of Mg^2+^.

The separation factor of lithium and magnesium (SF_Li-Mg_) and lithium recovery are shown in Figure 3d. It can be seen that the SF_Li-Mg_ rose from 15.4 to 30.1 in the first 30 min, and then gradually decrease from 30.1 to 22.8 in the next 75 min. The increasing concentration of Mg^2+^ in the collected retentate is unhelpful for the separation of lithium and magnesium. In addition, the lithium recovery increased almost linearly, and reached 91.6% at the end of the NF process. Noteworthily, the total salinity in the retentate liquid is too high, and the residual lithium cannot be directly recycled by NF, but this retentate liquid can be returned to the EID system to separate lithium and magnesium, which can reduce the waste of lithium.

The final compositions of the permeate flow produced by NF are shown in Table 5. From Table 5, it can be seen that the major cationic ions in the permeate flow are Li^+^, Na^+^, and Mg^2+^, and the main anionic ion is Cl^−^. The concentration of K^+^ is only 0.03 g·L^−1^, and other impurities such as Ca^2+^ and SO_4_^2−^ can almost be ignored.

#### 3.2.2. RO Process

**Figure 4:**
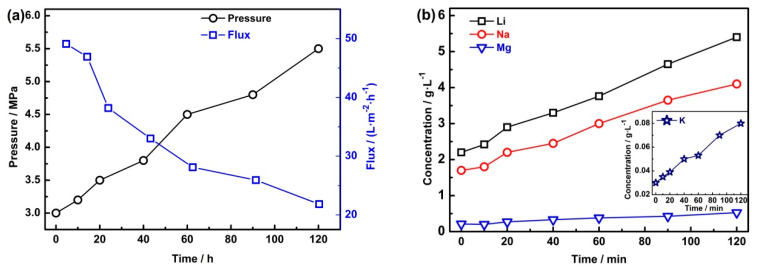

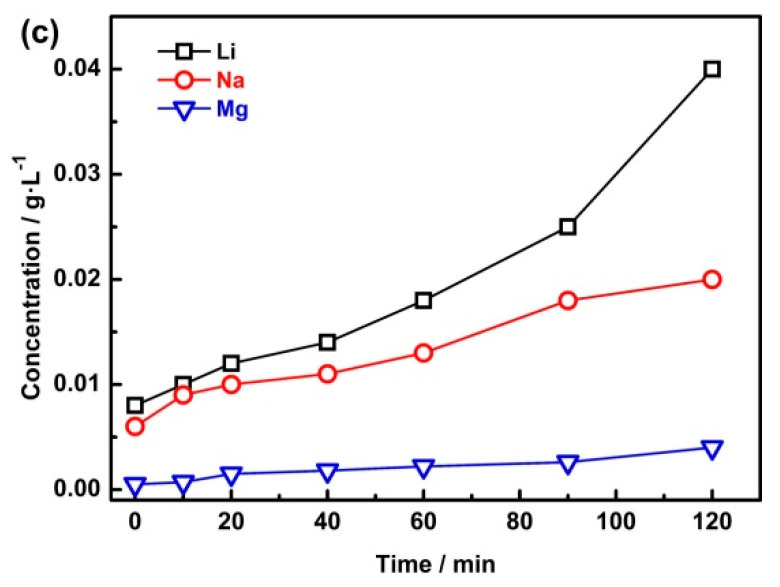
The main results in the RO process. (**a**) The relationship of the operation pressure and the flux of the membrane; **(b**) concentration of Li^+^, Na^+^, K^+^, and Mg^2+^ in the collected retentate; (**c**) concentration of Li^+^, Na^+^, K^+^, and Mg^2+^ in the permeate flow.

The permeate flow produced by the NF process was treated by the RO process, and the main results are shown in Figure 4. Figure 4a has shown that the operation pressure increased from the initial 3 MPa to 5.5 MPa during the RO process, while the flux of the water decreased from 49 L·m^−2^·h^−1^ to 21.8 L·m^−2^·h^−1^. Figure 4b shows that the concentration of ions such as Li^+^, Na^+^, Mg^2+^, and K^+^ in the collected retentate increased almost linearly. Concretely, Li^+^ has increased from 2.2 g·L^−1^ to 5.4 g·L^−1^ and Mg^2+^ increased from 0.21 g·L^−1^ to 0.525 g·L^−1^. Figure 4c shows that the concentration of Li^+^, Na^+^, and Mg^2+^ in the permeate flow increased significantly with the concentration process, but the maximum concentration of lithium is still lower than 0.04 g·L^−1^, and the lithium loss is almost negligible.

The final composition of the permeate flow and the collected retentate produced by RO is shown in Table 6. As shown in Table 6, the concentration of ions in the permeate flow is very low, the loss of the lithium in the RO permeate flow almost can be ignored, and the recovery of lithium can reach 99.4%. Moreover, the permeate flow with such a low salinity content can be used to prepare the supporting electrolyte for the EID system.

### 3.3. Precipitation of Li_2_CO_3_

The concentrations of Li^+^ and Mg^2+^ after RO are 5.4 g·L^−1^ and 0.525 g·L^−1^, respectively. This solution cannot be used directly for the precipitation of Li_2_CO_3_, and generally requires evaporation and impurity removal. Subsequently, we use an electric furnace to evaporate 5 L of solution to 1.2 L, and add NaOH to adjust the pH of the solution to 12.5 for further removal of magnesium (Mg^2+^ precipitates in the form of Mg(OH)_2_ when the solution is alkaline). The composition of the solution after magnesium removal is shown in Table 7.

As shown in Table 7, the concentration of Li^+^ is enriched to 21.6 g·L^−1^; the mass ratio of Na/Li is slightly greater than 1; and other ions such as K^+^, Mg^2+^, Ca^2+^, and SO_4_^2−^ are very low. The recovery of lithium in this process can reach 96.1%; such a low lithium loss is attributed to the effective removal of magnesium by the NF, which greatly reduces the generation of Mg(OH)_2_ and improves the recovery rate of lithium. In the actual production process, the water generated by evaporation can also be returned to the EID system to prepare the supporting electrolyte.

The solution with 21.6 g·L^−1^ lithium was used for the precipitation of Li_2_CO_3_ with 280 g·L^−1^ Na_2_CO_3_. Moreover, the concentration of the mother liquor is shown in Table 8. From Table 8, it can be seen that the main ions in the mother liquor are Na^+^ and Li^+^. Noteworthily, only 86.7% lithium was precipitated by Na_2_CO_3_, and the concentration of lithium in the mother liquor is still 1.8 g·L^−1^. In the same way, the mother liquor contains a small amount of excess carbonate, which can be neutralized by part of the brine with high Mg^2+^ ions, and then the mother liquor is returned to the EID system to recover the residual lithium.

**Figure 5:**
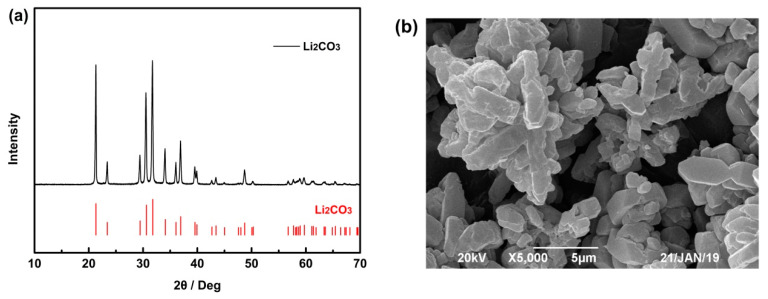
(**a**) XRD and (**b**) scanning electron microscope (SEM) of the obtained solid by precipitation.

The phase and morphology analysis of the obtained solid is shown in Figure 5. From Figure 5a, it can be seen that the XRD pattern of the obtained powder is indexed to Li_2_CO_3_ (JCPDS card 22-1141). Morphology analysis by SEM, as shown in Figure 5b, indicated that the particles were columnar and rod, mostly clusters, and have a relatively flat surface. The chemical composition of the prepared Li_2_CO_3_ is shown in Table 9, and the composition of the obtained Li_2_CO_3_ meets the national standard (Li_2_CO_3_-0, GB/T 11075-2013).

In general, the direct recovery of lithium from the high Mg/Li brine to the production of Li_2_CO_3_ can reach 68.7%, which was calculated by the product of the recovery of lithium in each process; considering that most of the solutions are cycled in the system (except the lithium loss by the precipitation of Mg(OH)_2_), the total recovery of lithium will be greater than 85%.

### 3.4. Comparison of Methods for Lithium Extraction from High Mg/Li Brine

Table 10 shows the comparison of methods for lithium extraction from high Mg/Li brine.

From Table 10, it can be seen that the total Li^+^ recovery rate in this paper is superior to that of ion sieve method and electrolysis method, but slightly lower than that of solvent extraction method. However, the extractant used in the solvent extraction method has a slight dissolution in the brine, which will cause greater environmental pollution. Noteworthily, this comprehensive membrane process has environmental protection significance.

## 4. Conclusions

In this paper, we constructed an integrated membrane process combining the EID system and NF and RO processes to prepare Li_2_CO_3_ from a high mass ratio of Mg/Li brine. This method successfully realizes the separation of lithium and magnesium in brine with a high Mg/Li ratio, which relies on the anion membrane to retain cations and the selective characteristics of LiFePO_4_ to adsorb lithium. Most of the bivalent ions in the prepared lithium-riched solution were removed by nanofiltration membrane. After concentration, purification, and precipitation, we prepared industrial-grade Li_2_CO_3_. Noteworthily, the removal of magnesium by nanofiltration can reduce the amount of alkali and reduce the entrainment loss of lithium caused by the massive production of magnesium hydroxide. In general, this process can efficiently realize the selective separation of magnesium and lithium without pollution to the environment and provide a new perspective for extracting lithium from salt lakes.

## Figures and Tables

**Table 1:** The components of the West Taijnar Salt Lake brine (g·L^−1^).

Element	Li^+^	Na^+^	K^+^	Mg^2+^	Ca^2+^	SO_4_^2−^	Cl^−^
West Taijnar	2.05	0.81	0.48	120.56	0.04	31.01	360.9

**Table 2:** The concentration of the main ions in the obtained anolyte (g·L^−1^).

Components	Li^+^	Na^+^	K^+^	Mg^2+^	Ca^2+^	SO_4_^2−^	Cl^−^
Concentration	2.1	1.9 *	0.04	1.95	0.004	0.26	19.1
Recovery & Rejection %	90.6	-	92.2	98.5	-	99.2	-

* The initial concentration of Na^+^ added in the form of NaCl is 2.0 g·L^−1^.

**Table 3:** The main analytical results in the collected retentate during the nanofiltration (NF) process.

Time	Conductivity	T	Flow		Concentration of Ions/g·L^−^^1^					
min	ms·cm^−1^	°C	L·min^−1^	Li^+^	Na^+^	K^+^	Mg^2+^	Ca^2+^	Cl^−^	SO_4_^2−^
0	37.5	24.7	16	2.10	1.91	0.04	1.95	0.004	19.1	0.26
30	40.9	25.3	16	2.26	1.82	0.038	3.64	0.0053	24.5	0.35
60	44.9	26.2	16	2.32	1.91	0.037	5.34	0.0085	30.2	0.56
90	52.0	27.0	16	2.28	1.69	0.036	9.78	0.018	42.1	1.21
105	108.2	27.6	16	2.31	1.96	0.041	18.23	0.02	66.8	2.37

**Table 4:** The solubility of all the chloride and sulfate exist in the collected retentate.

Compound	LiCl	NaCl	KCl	MgCl_2_	CaCl_2_ *	Li_2_SO_4_	Na_2_SO_4_	CaSO_4_ *	MgSO_4_	K_2_SO_4_
solubility/g	83.5	35.9	34.2	54.6	74.5	34.8	19.5	0.255	33.7	11.1

* The solubility of calcium chloride and calcium sulfate refers to the solubility of their hydrated salts; they are CaCl_2_·6H_2_O and CaSO_4_·2H_2_O, respectively.

**Table 5:** The compositions of the permeate flow produced by NF (g·L^−1^).

Elements	Li^+^	Na^+^	K^+^	Mg^2+^	Ca^2+^	Cl^−^	SO_4_^2−^	Lithium Recovery %
Concentration	2.2	1.7	0.03	0.21	3.1 × 10^−4^	14.39	0.0013	91.6

**Table 6:** The final compositions of the permeate flow and collected retentate.

Elements	Li^+^	Na^+^	K^+^	Mg^2+^	Ca^2+^	SO_4_^2−^	Lithium Recovery %
Permeate flow	0.021	0.011	4 × 10^−4^	0.002	/	/	/
Collected retentate	5.4	4.4	0.08	0.525	6.3 × 10^−4^	0.003	99.4

**Table 7:** The composition of the solution after magnesium removal (g·L^−1^).

Elements	Li^+^	Na^+^	K^+^	Mg^2+^	Ca^2+^	SO_4_^2−^	Lithium Recovery %
Concentration	21.6	23.9	0.34	0.002	2.9 × 10^−4^	0.018	96.1

**Table 8:** The main parameters of the mother liquor (g·L^−1^).

Element	Li^+^	Na^+^	K^+^	Lithium Recovery %
Concentration	1.8	90	0.2	86.7

**Table 9:** The chemical composition of the prepared Li_2_CO_3_.

Constituents	Li_2_CO_3_	Na	Mg	Fe	Ca	SO_4_^2−^	Cl^−^
Content (%)	99.6	0.026	0.005	0.0013	0.011	0.007	0.012

**Table 10:** Comparison of the Li^+^ recovery between this study and conventional methods.

Methods	Li^+^ Concentration in Brine/g·L^−1^	Mg/Li in Brine	Li^+^ Recovery Rate %	References
Solvent extraction	2.088	44.06	90.93 *	[39]
Ion sieve	0.259	95	82.1 *	[40]
Electrodialysis	0.148	60	72.1 *	[41]
This study	2.05	58.5	>85	This study

* The asterisk only indicates the recovery rate of the separation of magnesium and lithium from brine.
